# Supplementary material for: Spatial Variation in Stream-Water Bacterial Communities Across Sites Differing in Wild Chinese Giant Salamander Detection Frequency
Source: Biology (Basel). 2026 Sep 18;15(18):1647. doi: 10.3390/biology15181647 (PMC13604818; doi:10.3390/biology15181647)
Supplement: Supplementary file 1 [file biology-15-01647-s001.zip › Data Related to “Spatial Variation in Stream-Water Bacterial Communities across Sites Differing in Wild Chinese Giant Salamander Detection Frequency”/Microbial Data/result/08.FunctionPrediction/picrust2/heatmap/sample/heatmap.sample.TIGRFAM.cluster.pdf]

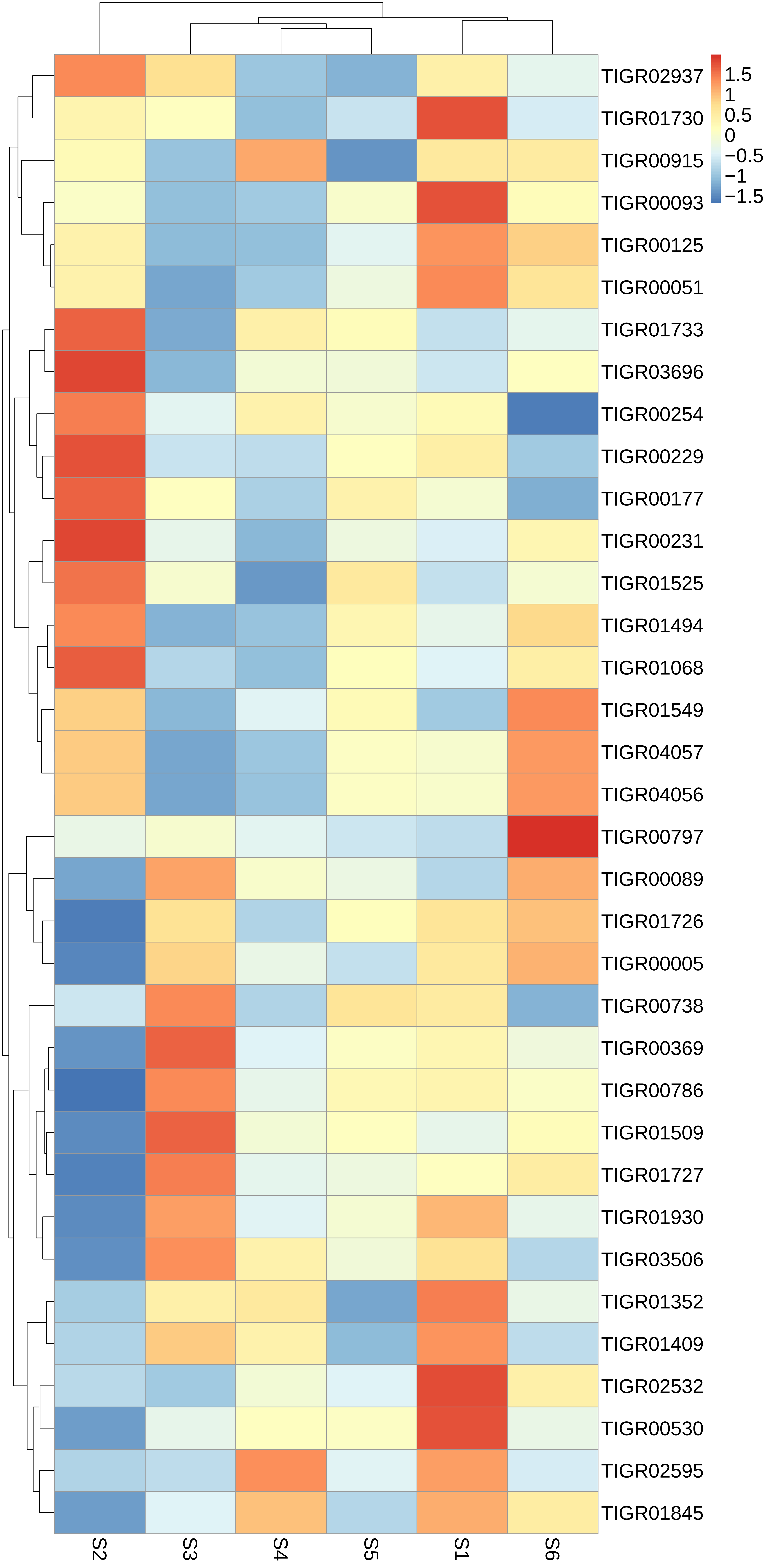

1.5
1
0.5
0
−0.5
−1
−1.5
TIGR02937
TIGR01730
TIGR00915
TIGR00093
TIGR00125
TIGR00051
TIGR01733
TIGR03696
TIGR00254
TIGR00229
TIGR00177
TIGR00231
TIGR01525
TIGR01494
TIGR01068
TIGR01549
TIGR04057
TIGR04056
TIGR00797
TIGR00089
TIGR01726
TIGR00005
TIGR00738
TIGR00369
TIGR00786
TIGR01509
TIGR01727
TIGR01930
TIGR03506
TIGR01352
TIGR01409
TIGR02532
TIGR00530
TIGR02595
TIGR01845
S2
S3
S4
S5
S1
S6
